# Ovarian Cancer Gene Therapy Using HPV-16 Pseudovirion Carrying the HSV-tk Gene

**DOI:** 10.1371/journal.pone.0040983

**Published:** 2012-07-17

**Authors:** Chien-Fu Hung, An Jen Chiang, Hsiao-Hsuan Tsai, Martin G. Pomper, Tae Heung Kang, Richard R. Roden, T.-C. Wu

**Affiliations:** 1 Department of Pathology, Johns Hopkins Medical Institutions, Baltimore, Maryland, United States of America; 2 Department of Oncology, Johns Hopkins Medical Institutions, Baltimore, Maryland, United States of America; 3 Department of Obstetrics and Gynecology, Kaohsiung Veterans General Hospital, Kaohsiung, Taiwan; 4 Division of Obstetrics and Gynecology, National Yang-Ming University School of Medicine, Taipei, Taiwan; 5 Department of Biological Sciences, National Sun Yat-Sen University, Kaohsiung, Taiwan; 6 Department of Radiology, Johns Hopkins School of Medicine, Baltimore, Maryland, United States of America; 7 Department of Obstetrics and Gynecology, Johns Hopkins Medical Institutions, Baltimore, Maryland, United States of America; 8 Department of Molecular Microbiology and Immunology, Johns Hopkins Medical Institutions, Baltimore, Maryland, United States of America; City of Hope National Medical Center and Beckman Research Institute, United States of America

## Abstract

Ovarian cancer is the leading cause of death from all gynecological cancers and conventional therapies such as surgery, chemotherapy, and radiotherapy usually fail to control advanced stages of the disease. Thus, there is an urgent need for alternative and innovative therapeutic options. We reason that cancer gene therapy using a vector capable of specifically delivering an enzyme-encoding gene to ovarian cancer cells will allow the cancer cell to metabolize a harmless prodrug into a potent cytotoxin, which will lead to therapeutic effects. In the current study, we explore the use of a human papillomavirus (HPV) pseudovirion to deliver a herpes simplex virus thymidine kinase (HSV-tk) gene to ovarian tumor cells. We found that the HPV-16 pseudovirion was able to preferentially infect murine and human ovarian tumor cells when administered intraperitoneally. Furthermore, intraperitoneal injection of HPV-16 pseudovirions carrying the HSV-tk gene followed by treatment with ganciclovir led to significant therapeutic anti-tumor effects in murine ovarian cancer-bearing mice. Our data suggest that HPV pseudovirion may serve as a potential delivery vehicle for ovarian cancer gene therapy.

## Introduction

Ovarian cancer is the leading cause of death from all gynecological cancers and the sixth most common malignancy for women in the United States [1], [2]. Although significant advances have occurred in both surgical and chemotherapeutic techniques, the overall 5-year survival rates for all stages of ovarian cancer remain <50% [2], [3]. Current therapies (surgery, chemotherapy, and radiotherapy) usually fail to control advanced stages of the disease. Therefore, alternative therapeutic approaches may serve as important methods for controlling these advanced stage ovarian tumors.

One possible approach is the use of suicide gene therapy (SGT). With SGT, the gene for a foreign enzyme (i.e. one from a virus, bacteria, or yeast) is specifically delivered to cancer cells. Gene delivery is followed by systemic administration of a nontoxic prodrug. The infected cancer cells are able to express the foreign enzyme to convert the prodrug into an active cytotoxin, which is able to kill the infected cells. One advantage SGT has over conventional gene therapies is its ability to kill neighboring cells through the bystander effect. The active drug can escape the transduced cells and diffuse into neighboring non-infected cells, ultimately leading to their death as well. The dying cells are also able to induce natural killer (NK) cells and T cells to induce a distant bystander effect. The hope for this approach is to have great specificity for tumor cells, particularly cancer stem cells. This approach should also reduce the toxic side effects associated with conventional cancer therapies due to the increased specificity of both the delivery and activation of the cytotoxin [4].

The most studied suicide gene/prodrug system is the combination of herpes simplex virus thymidine kinase (HSV-tk) with ganciclovir (GCV) [5]. HSV-tk, which has high affinity for ganciclovir, catalyzes the first phosphorylation of GCV that can then be di- and tri-phosphorylated by cellular kinases. Triphosphorylated GCV can be incorporated into replicating DNA, which leads to polymerase inhibition and eventually apoptosis [4]–[6]. This system has shown some success in the clinic but its utility is limited by its dependence on cell-to-cell contact and gap junctions for the bystander effect [4].

**Figure 1:**
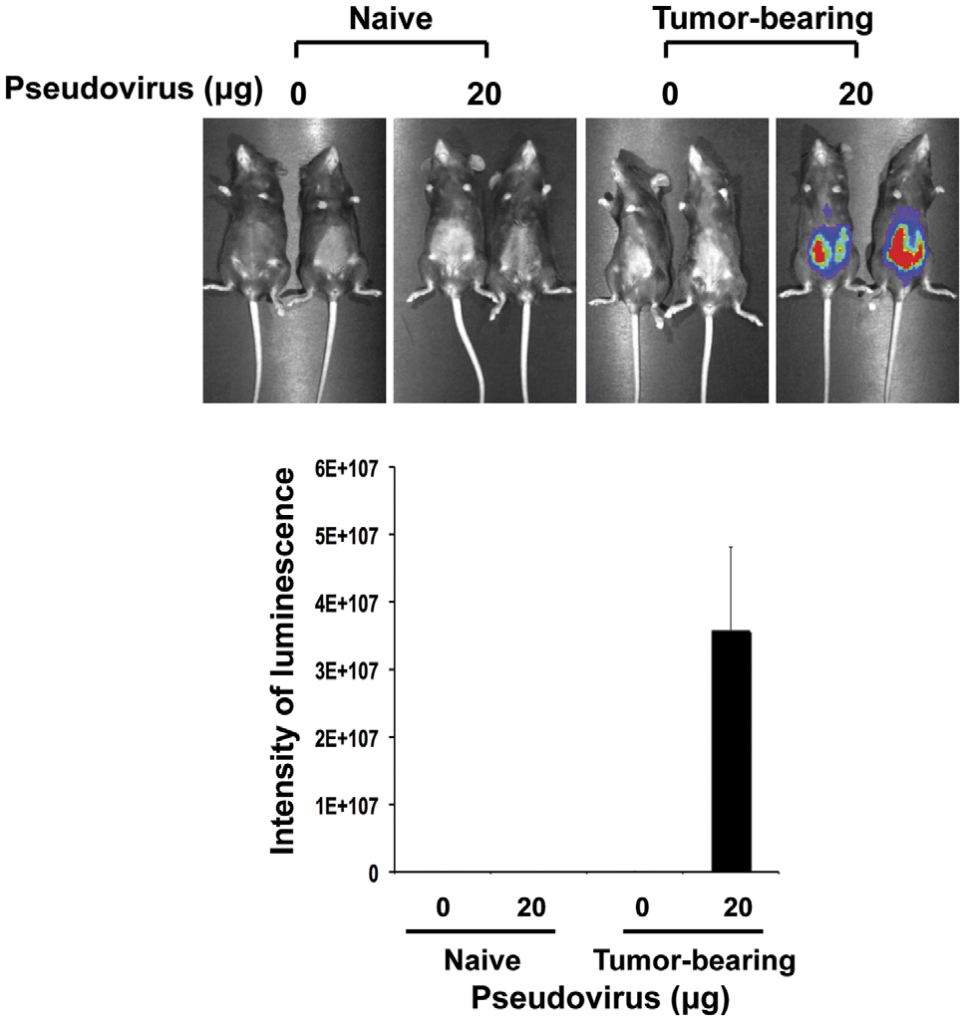
Comparison of HPV pseudovirion infectivity in naïve and murine ovarian tumor-bearing mice. 5–8 weeks old C57BL/6 mice were challenged with MOSEC tumor cells (1×10^6^ cells/mouse). One week later, tumor-bearing mice were intraperitoneally injected with or without HPV-16/luc pseudovirions (20 µg HPV-16 L1 protein/mouse, equivalent to 120 ng of DNA/mouse). Naïve mice infected with or without HPV-16/luc pseudovirions served as a control. Mice were imaged by non-invasive luminescence imaging 1 day after infection. Data shown are representative of 2 experiments performed.

For this study, we use replication-defective human papillomavirus (HPV) pseudovirions to deliver the HSV-tk gene to ovarian tumor cells. Recent studies show DNA plasmids can be packaged into the papillomavirus L1 and L2 capsid proteins to generate the ‘pseudovirion’ that can efficient deliver the DNA into multiple cell lines [7]–[9]. The encapsulation also protects the DNA from nucleases and provides a targeted delivery with great stability. Many of the safety concerns associated with the use of live viral vectors are alleviated as the HPV pseudovirions contain a DNA construct different from the natural HPV viral genome. There are also over 100 types of papillomavirus pseudovirions, which allows for repeated boosting using different types since the neutralizing antibodies against one type are usually not cross-reactive with other types.

Here we explore the use of HPV pseudovirions to deliver marker genes and suicidal genes to both murine and human ovarian tumor cells. We found that intraperitoneal injection of HPV-16 pseudovirions led to preferential infection of ectopic and spontaneously occurring murine ovarian cancers in mice. Preferential infection also occurred in human ovarian tumor xenografts of tumor-bearing mice. Intraperitoneal injection of HPV-16 pseudovirions carrying the HSV-tk gene followed by administration of ganciclovir led to significant therapeutic anti-tumor effects in murine-ovarian cancer-bearing mice. Our data shows proof-of-principle that HPV pseudovirions can be useful vectors for delivering therapeutic genes to ovarian cancers.

**Figure 2:**
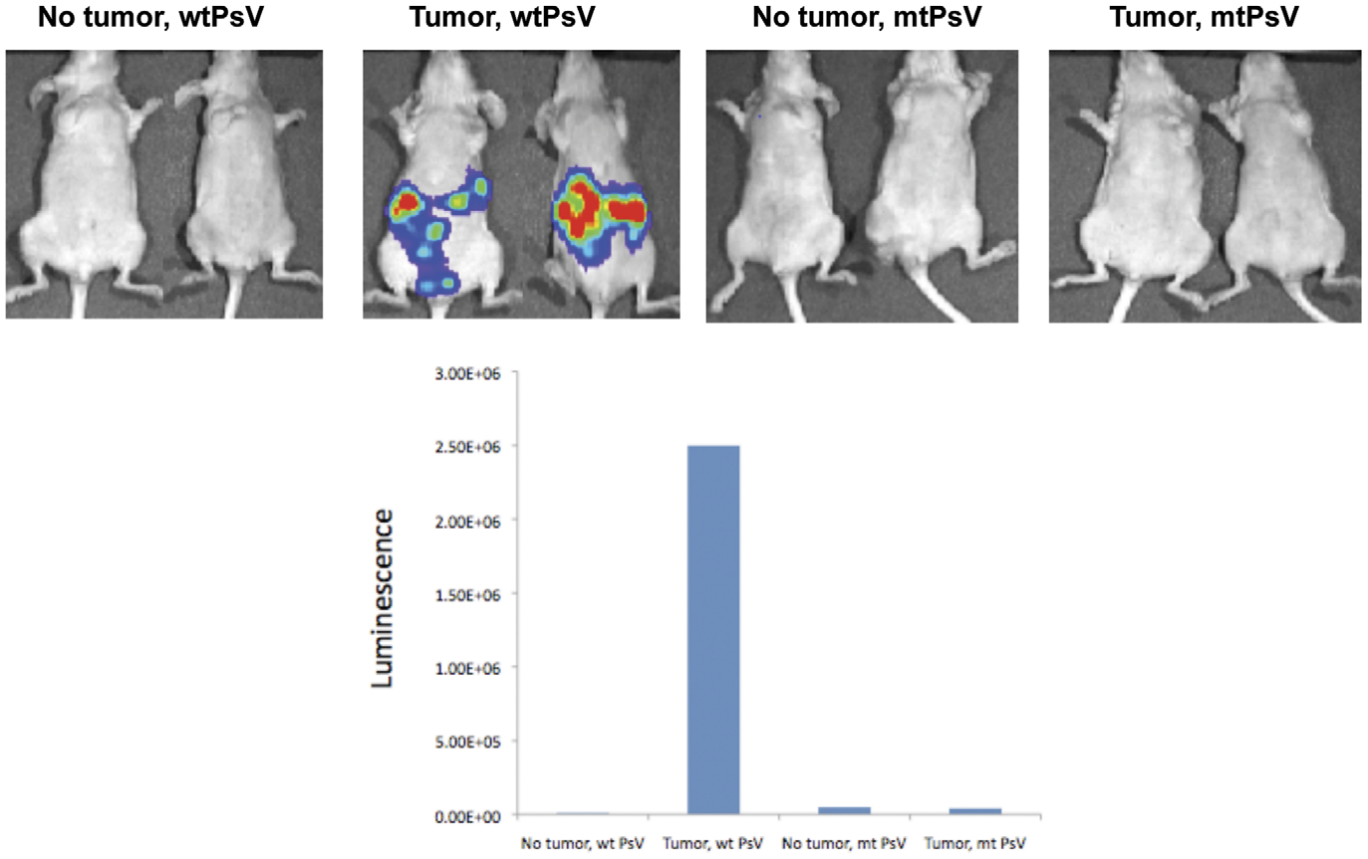
Characterization of the infectivity of HPV pseudovirion in naïve nude mice and human ovarian tumor-bearing nude mice. 5–8 weeks old nude mice were injected intraperitoneally with ES2 human ovarian tumor cells (1×10^6^ cells/mouse). One week later, tumor-bearing mice were intraperitoneally injected with wild-type (wt) HPV-16/Luc psV or mutant L2 (mtL2) HPV-16L1mtL2-Luc pseudovirions (20 µg HPV-16 L1 protein/mouse, equivalent to 120 ng of DNA/mouse). Naïve mice infected with wt or mt HPV-16/luc pseudovirions served as controls. Mice were imaged by non-invasive luminescence imaging 1 day after infection. Data shown are representative of 2 experiments performed.

## Materials and Methods

### Ethics Statement

This study was carried out in strict accordance with the recommendations of the Guide for the Care and Use of Laboratory Animals of the National Institutes of Health. All procedures were performed with prior approval of the Johns Hopkins Animal Care and Use Committee (protocol MO08M446).

### Mice

The C57BL/6 and nude (BALB/c nu/nu) mice were acquired from the National Cancer Institute. *MISIIR-TAG* transgenic mice [10] were kindly provided by Dr. Denise Connolly at the Fox Chase Cancer Center. All animals were maintained under specific pathogen-free conditions, and all procedures were performed according to approved protocols and in accordance with recommendations for the proper use and care of laboratory animals.

**Figure 3:**
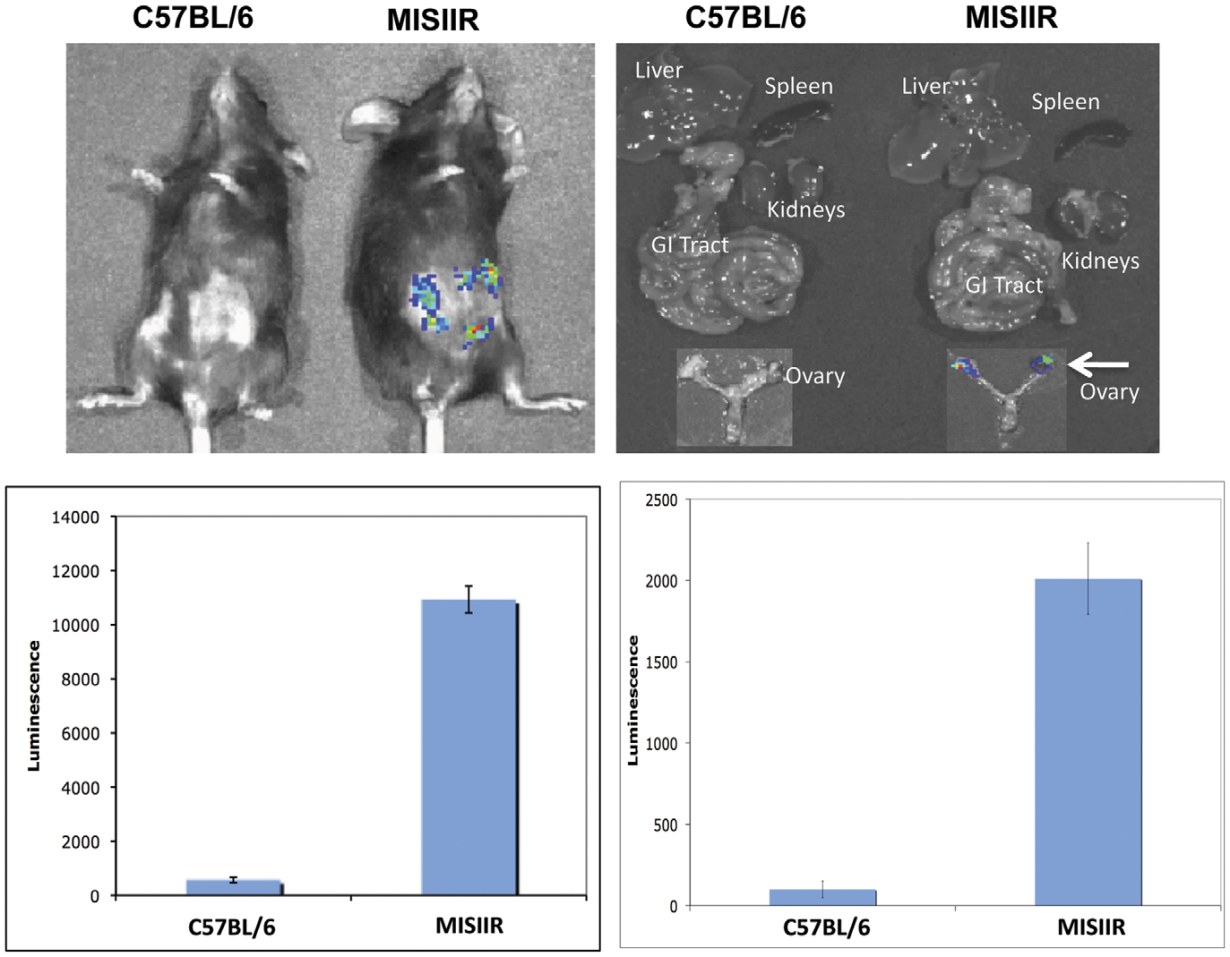
HPV-16/luc psV preferentially infects ovarian tumors in *MISIIR-TAG* transgenic mouse. C57BL/6 mice and *MISIIR-TAG* transgenic mice were injected with 20 µg of HPV-16/luc psV by intraperitoneal injection 10 weeks after birth. Luminescence images were taken 1 day after HPV-16/luv psV injection. *Top,* Representative luminescence images of psV-infected C57BL/6 mice or *MISIIR-TAG* transgenic mice (left) and their harvested organs (right). Note: White arrow indicates only ovarian cancer from MISRII transgenic mice can be infected by HPV-16/luc psV. *Bottom,* Representative bar graphs of luminescence imaging in MISIIR-TAG transgenic mice or C57BL/6 mice. Data representative of 2 experiments performed.

### Cell Lines

293TT cells were kindly provided by J Schiller (National Cancer Institute (NCI), National Institutes of Health (NIH)) [11]. The MOSEC cell line (a mouse ovarian cancer cell line) was prepared as described previously [12]. The ES2 cell line (a human ovarian cancer cell line) was purchased from the American Type Culture Collection (ATCC), Manassas, VA. MOSEC/luciferase (MOSEC-luc) cells were generated as described previously [13].

**Figure 4:**
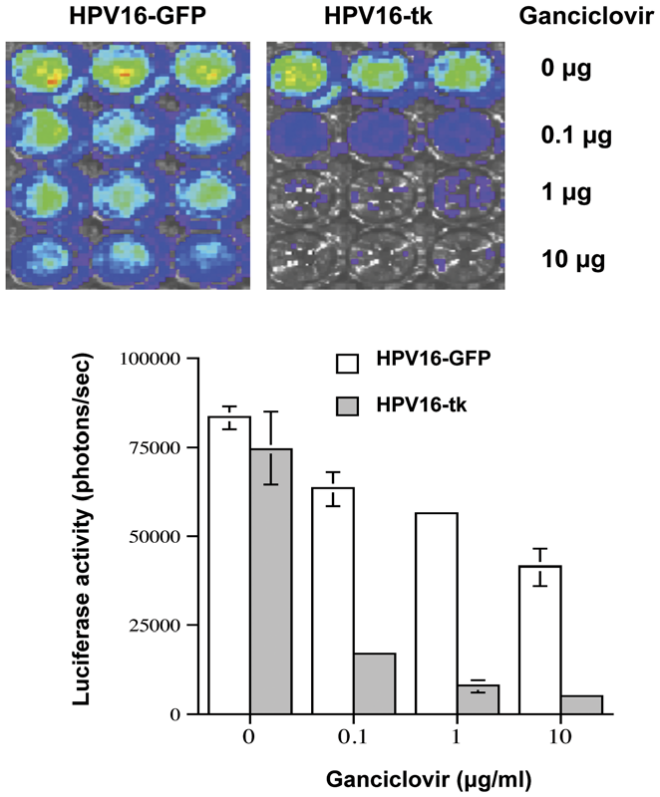
*In vitro* cytotoxicity mediated by HPV16-tk pseudovirions and ganciclovir. MOSEC-Luc cells were infected HPV16-GFP psV or HPV16/HSV-tk psV at a concentration of 1 µg L1 protein/ml for 48 hours. The infected cells were seeded in 96 well plates and then treated with 0 µg/ml, 0.1 µg/ml, 1 µg/ml, or 10 µg/ml of Ganciclovir for 72 hours. Luciferase expression was examined by the IVIS 200 system (Xenogen Corp., Alameda, CA, USA). Data shown are representative of 2 experiments performed.

### Plasmids

The plasmids encoding HPV16 L1 L2 (pShell16) were kindly provided by Dr. J Schiller (NCI). The point mutation HPV16L1 mtL2 construct is described in our previous study [14]. The generation of the luciferase-expressing plasmid (pcDNA3-luciferase) and the GFP-expressing plasmid (pcDNA3-GFP) has been described previously [15], [16]. The pORF-HSVtk plasmid was purchased from InvivoGen.

### HPV Pseudovirion Production

HPV16-GFP, HPV16-Luc (luciferase), HPV16-tk (HSVtk Herpes Simplex Virus-Thymidine Kinase) pseudovirions were made using the methods as described previously [11]. Briefly, 293TT cells were cotransfected with HPV expression plasmids pShell16 and the plasmids of choice (such as GFP, luciferase or HSVtk) using Lipofectamine 2000 (Invitrogen). After 48 h, the cells were harvested and washed with Dulbecco’s phosphate buffered saline (PBS) (Invitrogen) supplemented with 9.5 mM MgCl_2_ and antibiotic-antimycotic mixture (DPBS-Mg) (Invitrogen). The cells were suspended in DPBS-Mg supplemented with 0.5% Brij58, 0.2% Benzonase (EMD Chemicals, Gibbstown, NJ, USA), and 0.2% Plasmid Safe (Epicentre Biotechnologies, Madison, WI, USA) at >100×10^6^ cells/ml and incubated at 37 C for 24 h for capsid maturation. After maturation, the cell lysate was chilled on ice for 10 min. The salt concentration of the cell lysate was adjusted to 850 mM and incubated on ice for 10 min. The lysate was then clarified by centrifugation, and the supernatant was layered onto an Optiprep gradient. The gradient was spun for 4.5 h at 16 C at 40 000 r.p.m. in a SW40 rotor (Beckman Coulter, Inc., Brea, CA, USA). The purity of HPV pseudovirions was evaluated by running the fractions on 4–15% gradient sodium dodecyl sulfate-polyacrylamide gel electrophoresis. The encapsulated DNA plasmid was quantified by extracting encapsidated DNA from Optiprep factions followed by quantitative real-time PCR comparisons with serial dilutions of naked DNA. The concentrations of plasmids (GFP, luciferase, or HSVtk) in the pseudovirions were determined to be approximately 6.2 ng of DNA per 1 µg of L1 protein.

### *In vitro* Infection of Tumor Cells by HPV16 Pseudovirions

MOSEC or ES2 cells were plated in 96-well plates at a density of 5000 cells/well and grown overnight. The cells were infected with HPV-16/Luc psV (1 µg L1 protein/ml) for 72 hours. Luciferin (15 µg/ml) was added and incubated for 10 min. An integration time of 10 sec was used for luminescence image acquisition by the IVIS 200 system (Xenogen Corp., Alameda, CA, USA).

### Characterization of Tumor Infection by HPV16 Pseudovirions in Mice

C57BL/6 mice (5 per group) were intraperitoneally injected with 1×10^6^ MOSEC cells/mouse. One week later, tumor-bearing mice were intraperitoneally injected with HPV-16/Luc psV at a dose of 20 µg HPV-16 L1 protein/mouse (equivalent to 120 ng of DNA/mouse). Luminescence images were recorded the day after the HPV-16/Luc psV injection. An integration time of 2 min was used for luminescence image acquisition.

For the characterization of the infection of human ovarian cancer cells by HPV-16/Luc psV, nude mice were challenged with ES2 human ovarian tumor cells at a dose of 1×10^6^ cells/mouse. One week later, tumor-bearing mice were intraperitoneally injected with wild-type (wt) HPV-16/Luc psV or mutant (mt) HPV-16L1mtL2-Luc psV at a dose of 20 µg HPV-16 L1 protein/mouse (equivalent to 120 ng of DNA/mouse). Naïve mice without tumors infected with wt or mt HPV-16/Luc psV served as controls. Luminescence images were taken the day after the HPV-16/Luc psV injection. The mice were injected with 0.2 ml of 15 mg/ml beetle luciferin (potassium salt; Promega). After 10 min, the mice were imaged using the IVIS 200 system (Xenogen Corp., Alameda, CA, USA). An integration time of 30 sec was used for luminescence image acquisition.

### *In vitro* Cytotoxicity Mediated by HPV16-TK Pseudovirions and Ganciclovir

MOSEC-Luc cells were infected HPV16-GFP psV or HPV16-TK psV (1 µg L1 protein/ml) for 48 hours. The infected cells were seeded in 96 well plates. The infected cells were treated with 0 µg/ml, 0.1 µg/ml, 1 µg/ml, or 10 µg/ml of ganciclovir for 72 hours and luciferase expression was examined by IVIS 200 system (Xenogen Corp., Alameda, CA, USA).

### *In vivo* Cytotoxicity Mediated by HPV16-TK Pseudovirions and Ganciclovir

Mice were injected intraperitoneally with 1×10^6^ MOSEC-Luc cells/mouse on day 1. Luciferase activity was examined on day 2 as an indication of number of tumors in the mouse. The mice were injected with 0.2 ml of 15 mg/ml beetle luciferin (potassium salt; Promega). After ten minutes, the mice were imaged using the IVIS 200 system (Xenogen Corp., Alameda, CA, USA). An integration time of 2 min was used for luminescence image acquisition. Mice were injected with HPV16-GFP psV (20 µg L1 protein) or HPV16-TK psV (20 µg L1 protein) on day 3. Mice were treated daily with ganciclovir (50 mg/kg) or PBS from day 5 to day 18. Mice were imaged again by non-invasive luminescence imaging on day 20.

## Results

### Intraperitoneal Injection of HPV-16 Pseudovirions Leads to Preferential Infection of Murine Ovarian Cancer Cells in Tumor-bearing Mice

We initially examined if the HPV-16 pseudovirion carrying the luciferase gene (HPV-16/Luc psV) was capable of infecting the murine ovarian cancer cell line, MOSEC, *in vitro*. As shown in **Figure S1**, HPV-16/Luc psV was able to infect MOSEC tumor cells *in vitro*. To demonstrate if the HPV-16/Luc psV can also infect the murine ovarian cancer cell line in tumor-bearing mice, we first intraperitoneally injected mice with MOSEC tumor cells. The tumor-bearing mice were intraperitoneally injected with HPV-16/Luc psV one week later. As shown in **Figure 1**, while mice without tumors did not show any luciferase activity, tumor-bearing mice intraperitoneally injected with HPV-16/Luc psV demonstrated significant luciferase activity. These data suggest the HPV pseudovirion preferentially infects the tumor cells in tumor-bearing mice.

### Intraperitoneal Injection of HPV-16 Pseudovirions Leads to Preferential Infection of Human Ovarian Cancer Cells in Tumor-bearing Nude Mice

We further examined whether the HPV-16/Luc psV was able to infect the human ovarian cancer cell line ES2. As shown in **Figure S2**, ES2 cells infected with HPV-16/Luc psV demonstrated luciferase activity, suggesting that HPV-16/Luc psV was capable of infecting human ovarian cancer cells *in vitro*. We also characterized the *in vivo* infectivity of HPV-16/luc psV in nude mice bearing ES2 human ovarian tumors to determine whether the infectivity of HPV pseudovirions is essential for efficient gene delivery to ovarian tumor cells by HPV pseudovirions. It is now clear that the HPV L2 minor capsid protein is essential for efficient infection of cells by HPV pseudovirions [14], [17]. Thus, we have employed HPV-16/Luc psV with a single amino acid mutation in HPV L2 (HPV16L1mtL2-luc psV) that abolishes the infectivity of pseudovirions [14]. As shown in **Figure 2**, only nude mice bearing human ovarian tumors demonstrated significant luminescence compared to the non-tumor-bearing nude mice. Furthermore, only tumor-bearing mice infected by HPV-16/luc psV but not mutant HPV-16/L1mtL2- luc psV demonstrated significant luminescence (**Fig. 2**). Thus, our data show that HPV-16 pseudovirions can also preferentially infect human ovarian tumor cells *in vivo*. Furthermore, our data indicate that intact HPV L2 is essential for efficient delivery of encapsidated genes to ovarian tumor cells by HPV pseudovirions.

### HPV-16/luc psV Preferentially Infects Ovarian Tumors in *MISIIR-TAg* Transgenic Mouse

We further examined the preferential infection of ovarian tumor cells by HPV pseudovirions in the *MISIIR-TAg* transgenic mouse, a spontaneously occurring murine ovarian tumor model. This transgenic mouse, which expresses the transforming region of SV40 under the control of the Mullerian inhibitory substance type II receptor gene promoter, has been shown to develop bilateral ovarian tumors. Thus, the spontaneous ovarian cancer model in *MISIIR-TAg* transgenic mice more closely resembles human ovarian cancer than a transplantation model such as MOSEC tumor model. The *MISIIR-TAg* transgenic mouse has been reported to spontaneously develop ovarian carcinoma within 6–13 weeks of birth [10]. In our lab, all of the *MISIIR-TAg* transgenic mice (from a new line acquired from Dr. Connolly) developed ovarian tumors within 4 months of birth. We injected HPV-16/luc psV 10 weeks after birth and we found that HPV-16/Luc psV could also preferentially infect the spontaneously occurring ovarian cancer in the *MISIIR-TAg* transgenic mice (**Fig. 3**). Furthermore, we observed that the vital organs including the lungs, heart, stomach, spleen and kidney were not infected. In addition, no luciferase activity was observed in the normal ovaries of C57BL/6 mice injected with HPV-16/luc psV. Thus, our data indicate that HPV-16 pseudovirions can selectively infect ovarian tumor cells in a spontaneously occurring murine ovarian tumor model.

### Infection of HPV-16 Pseudovirions Carrying the HSV-tk Gene followed by Treatment with Ganciclovir Leads to *in vitro* Cytotoxicity of Infected Cells

The preferential infection of HPV-16 pseudovirions in ovarian tumor cells allows for the opportunity to specifically carry therapeutic DNA to ovarian tumor cells for the control of ovarian tumors. Because the initial administration of HPV pseudovirions may not be able to infect all of the ovarian tumor cells, it is important to consider a strategy that allows the ovarian tumor cells not directly infected to also become susceptible. Therefore, we chose the HSV-tk/ganciclovir system, as it is the most widely studied suicide gene therapy system and over two decades of attempts to use the system as an anticancer therapy has shown varying success. To demonstrate if HPV-16/HSV-tk psV can infect ovarian tumor cells and render them susceptible to killing by treatment with ganciclovir, we infected MOSEC-Luc cells with HPV16-GFP psV or HPV16/HSV-tk psV for 48 hours. The infected cells were subsequently treated with different concentrations of ganciclovir and the luciferase expression of the infected cells was measured using the IVIS 200 system. As shown in **Figure 4**, MOSEC-Luc tumor cells infected with HPV-16/HSV-tk psV followed by treatment with ganciclovir led to cell death of the infected cells *in vitro.* This was not observed in cells infected with HPV-16/GFP. We also observed that higher concentrations of ganciclovir led to more cell death. Similar effects were seen when similar assays were conducted with the human ovarian cancer cell line ES2 (see **Figure S3**).

### Intraperitoneal Injection of HPV-16 Pseudovirions Carrying the HSV-tk Gene followed by Treatment with Ganciclovir Leads to Significant Antitumor Effects in Murine Ovarian Cancer-bearing Mice

**Figure 5:**
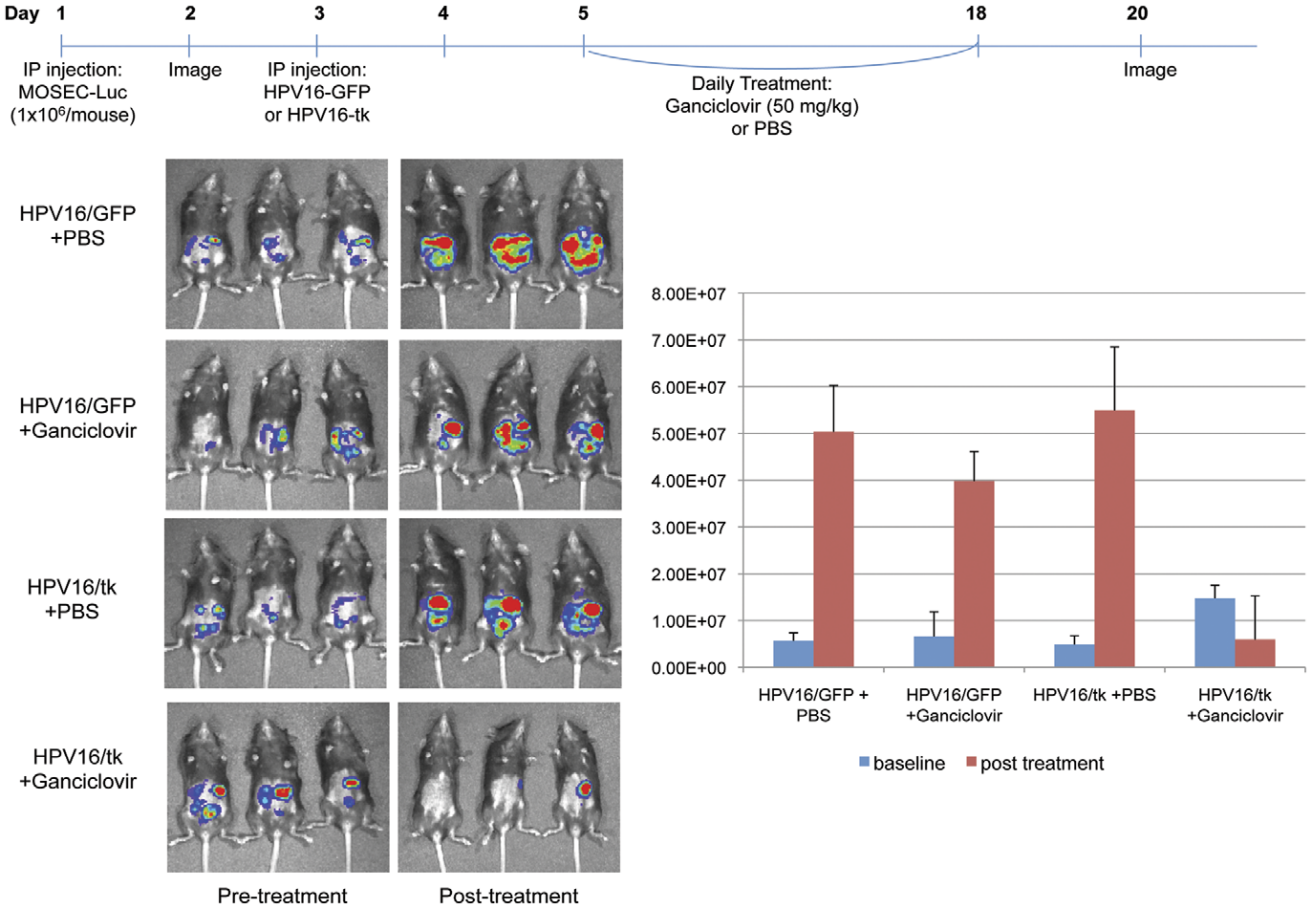
*In vivo* cytotoxicity mediated by HPV16-tk pseudovirions and ganciclovir. C57BL/6 mice (5 per group) were intraperitoneally injected with 1×10^6^ MOSEC-Luc cells per mouse on day 1. Luciferase activity was examined on day 2 as indication of number of tumors in the mice. Mice were injected HPV16-GFP psV (L1 protein 20 µg) or HPV16/HSV-tk psV at a dose of L1 protein (20 µg/mouse) on day 3. Mice were treated daily with ganciclovir at a dose of 50 mg/kg or PBS from day 5 to day 18. Mice were imaged by non-invasive luminescence imaging techniques on day 20. Data shown are representative of 2 experiments performed.

We further determined if MOSEC tumor-bearing mice infected with HPV-16/HSV-tk psV followed by treatment with ganciclovir could exhibit therapeutic antitumor effects. The mice were injected with MOSEC-Luc tumor cells and then treated with HPV-16/HSV-tk psV or HPV-16/GFP psV using regimen as described in **Figure 5**. The mice were subsequently treated with ganciclovir and tumor growth was monitored using a luminescence imaging system. As shown in **Figure 5**, tumor-bearing mice treated with HPV-16/HSV-tk psV followed by treatment with ganciclovir exhibited significantly better therapeutic antitumor effects than mice treated with HPV-16/GFP psV followed by treatment with ganciclovir. These data indicate HPV-16 pseudovirion can be used to effectively deliver the HSV-tk gene to ovarian tumor cells to render ovarian tumor cells more susceptible to treatment with ganciclovir.

## Discussion

The successful employment of the HSV-tk gene for ovarian cancer gene therapy using HPV pseudovirions suggests that other suitable candidate genes may also be delivered by HPV pseudovirion to ovarian tumors for ovarian cancer gene therapy. Several candidate genes for enzyme-prodrug combinations for suicide gene therapy have been reported including cytosine deaminase [18], [19], nitroreductase [20], carboxylesterase [21], cytochrome p450 [22] and purine nucleoside phosphorylase [23]. Like HSV-tk, the enzymes encoded by these genes can convert the nontoxic prodrugs into drugs capable of blocking DNA synthesis, resulting in eventual cell death as well as bystander effects to kill additional neighboring tumor cells.

For future clinical translation, it will be important to consider safety concerns of both the HSV-tk/GCV system and the delivery vehicle. However, cancer gene therapy using HSV-tk has been extensively used. Many clinical trials using this approach have been conducted in patients with gliomas and no serious adverse events were reported (for review see [24], [25]). On the other hand, production of the HPV pseudovirion as DNA delivery vehicles will likely require further developments to improve its safety profile. The current cell line used for generating the pseudovirions contains SV40 large T antigen, which is an oncoprotein, and therefore raises some possible safety concerns. An alternative approach using yeast to produce pseudovirions has been described [26]. The mass production of HPV pseudovirions will also likely require efficient standardized protocols to generate large titers of infectious HPV pseudovirions for clinical translation.

In the current study, we demonstrated preferential infection of murine and human ovarian tumors by HPV-16 pseudovirion. Our findings are consistent with previous studies by Roberts et al. using a nude mouse model for peritoneal dissemination of human ovarian cancer cell line, SHIN3 [27]. They also found that HPV pseudovirion administered intraperitoneally infected ovarian tumor tissues with high specificity while skipping the normal peritoneal tissue surfaces. However, different types of HPV pseudovirion may demonstrate different tumor and/or tissue tropisms. For example, Cerio et al, have shown that an HPV-16, but not HPV-45, pseudovirus could infect SWA-G human ovarian tumor cells *in vitro* [28]. Their data suggest that pseudovirus infection of human tumors may be HPV type- and tumor-specific. Thus, it is important to further identify the right types of HPV pseudovirion for future cancer gene therapy.

The mechanisms for the preferential infection of the ovarian tumor cells by HPV pseudovirions remain unclear. In our study, we showed that an intact L2 is essential for the infectivity of HPV-16 pseudovirion in ovarian tumors (see **Figure 2**). HPV entry to target cells has been shown to be initiated by first binding to heparin sulfonated proteoglycan (HSPG) cell surface attachment factors. The HPV viral particles then undergo a conformational change that exposes the N-terminus of L2 minor capsid protein to furin cleavage [29], [30]. The proteolysis of L2 exposes a previously occluded surface of L1 that binds to a currently undetermined cell surface receptor on cells, which is believed to be responsible for particle internalization (for review see [31]). It has also been shown that most ovarian cancer cell lines upregulate furin expression [32]. Thus, it is conceivable that the upregulation of furin expression may contribute to the preferential infection of the ovarian tumor by HPV pseudovirions. We can also exclude the possibility that preferential infection is an artifact of passaging the tumor cells *in vitro* since preferential infection was observed in the spontaneously occurring ovarian tumor model (see **Figure 3**). It will be important to further characterize the mechanisms for the preferential infection of the ovarian tumor cells by HPV pseudovirions. Such information will be useful for improving the specific delivery of gene of interest to ovarian tumor cells using HPV pseudovirions.

In summary, we found that intraperitoneal injection of HPV-16 pseudovirions leads to preferential infection of murine and human ovarian cancer cells in tumor-bearing mice. Furthermore, cancer gene therapy using HPV pseudovirions carrying HSV-tk was capable of specifically targeting the ovarian tumors, resulting potent therapeutic antitumor effects against ovarian tumors. It will be important to further identify the most suitable gene therapy candidates and therapeutic regimens for future studies towards clinical translation.

## Supporting Information

Figure S1 **Characterization of the infection of MOSEC tumor cells by HPV-16 pseudovirions** ***in vitro*** **.** MOSEC cells were seeded into 96-well plates at a density of 5,000 cell/well the night before infection. The seeded MOSEC cells were then infected with HPV-16/Luc psV (1 µg L1 protein/ml) for 72 hours and luciferase expression was examined by the IVIS 200 system (Xenogen Corp., Alameda, CA, USA). Luminescence images of MOSEC ovarian cancer cell line (top). Bar graph depicting the total photon counts for each well (bottom). Note: HPV-16 pseudovirions can efficiently infect MOSEC mouse ovarian cancer *in vitro*. Data shown are representative of 2 experiments performed.
(TIF)

Figure S2 **Characterization of the infection of ES2 human ovarian cancer cells by HPV-16 pseudovirions** ***in vitro*** **.** ES2 human ovarian tumor cells were seeded into 96-well plates at a density of 5,000 cells/well the night before infection. The seeded ES2 cells were then infected with HPV-16/Luc psV (1 µg L1 protein/ml) for 72 hours and luciferase expression was examined by IVIS 200 system (Xenogen Corp., Alameda, CA, USA). Luminescence images of ES2 ovarian cancer cell line (top). Bar graph depicting the total photon counts for each well (bottom). Note: HPV-16 pseudovirions can efficiently infect ES2 human ovarian cancer in vitro. Data shown are representative of 2 experiments performed.
(TIF)

Figure S3 ***In vitro*** **cytotoxicity mediated by HPV16-tk pseudovirions and ganciclovir.** ES2-Luc cells were infected HPV16-GFP psV or HPV16/HSV-tk psV at a concentration of 1 µg L1 protein/ml for 48 hours. The infected cells were seeded in 96 well plates and then treated with 0 µg/ml, 0.1 µg/ml, 1 µg/ml, or 10 µg/ml of Ganciclovir for 72 hours. Luciferase expression was examined by the IVIS 200 system (Xenogen Corp., Alameda, CA, USA). Data shown are representative of 2 experiments performed.
(TIF)
