# Supplementary figures and images for: Ovarian Cancer Gene Therapy Using HPV-16 Pseudovirion Carrying the HSV-tk Gene
Source: PLoS One. 2012 Jul 17;7(7):e40983. doi: 10.1371/journal.pone.0040983 (PMC3398866; doi:10.1371/journal.pone.0040983)

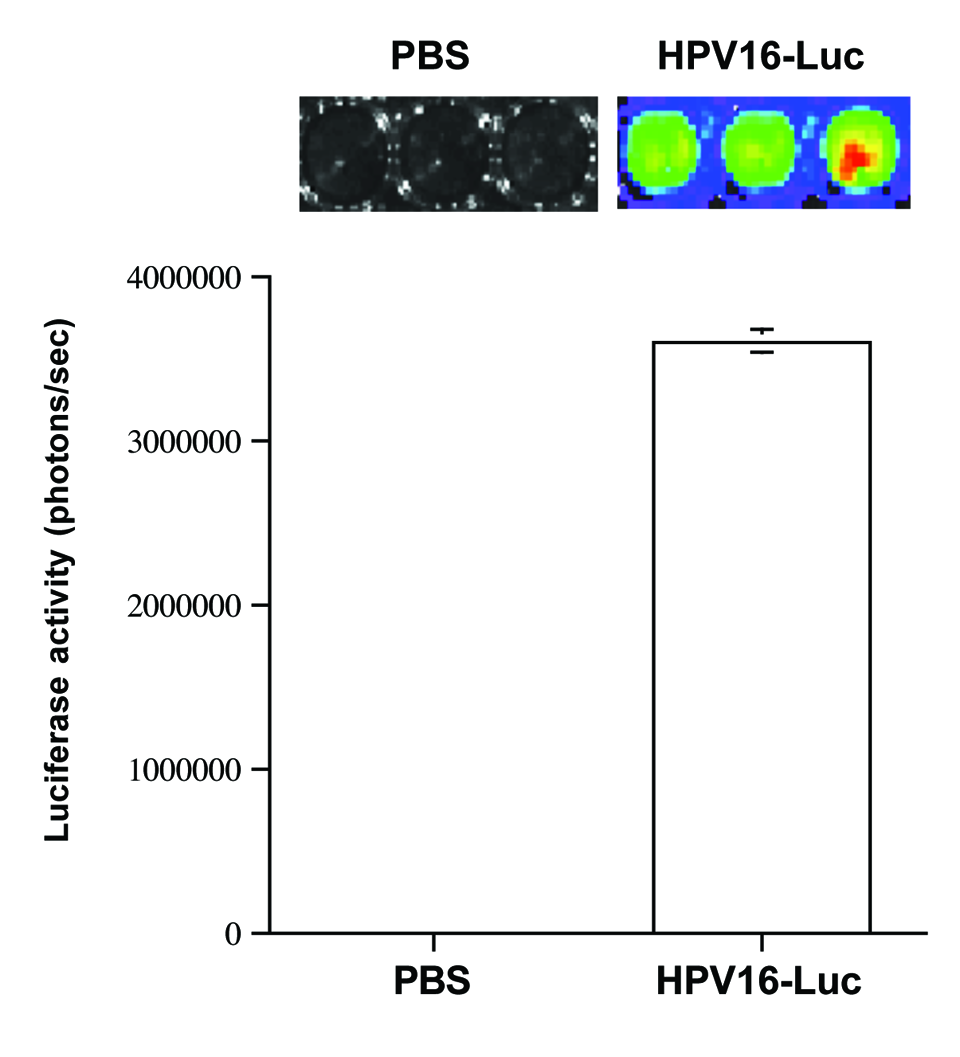

Supplement: Figure S1 — Characterization of the infection of MOSEC tumor cells by HPV-16 pseudovirions in vitro . MOSEC cells were seeded into 96-well plates at a density of 5,000 cell/well the night before infection. The seeded MOSEC cells were then infected with HPV-16/Luc psV (1 µg L1 protein/ml) for 72 hours and luciferase expression was examined by the IVIS 200 system (Xenogen Corp., Alameda, CA, USA). Luminescence images of MOSEC ovarian cancer cell line (top). Bar graph depicting the total photon counts for each well (bottom). Note: HPV-16 pseudovirions can efficiently infect MOSEC mouse ovarian cancer in vitro. Data shown are representative of 2 experiments performed. (TIF) [file pone.0040983.s001.tif]

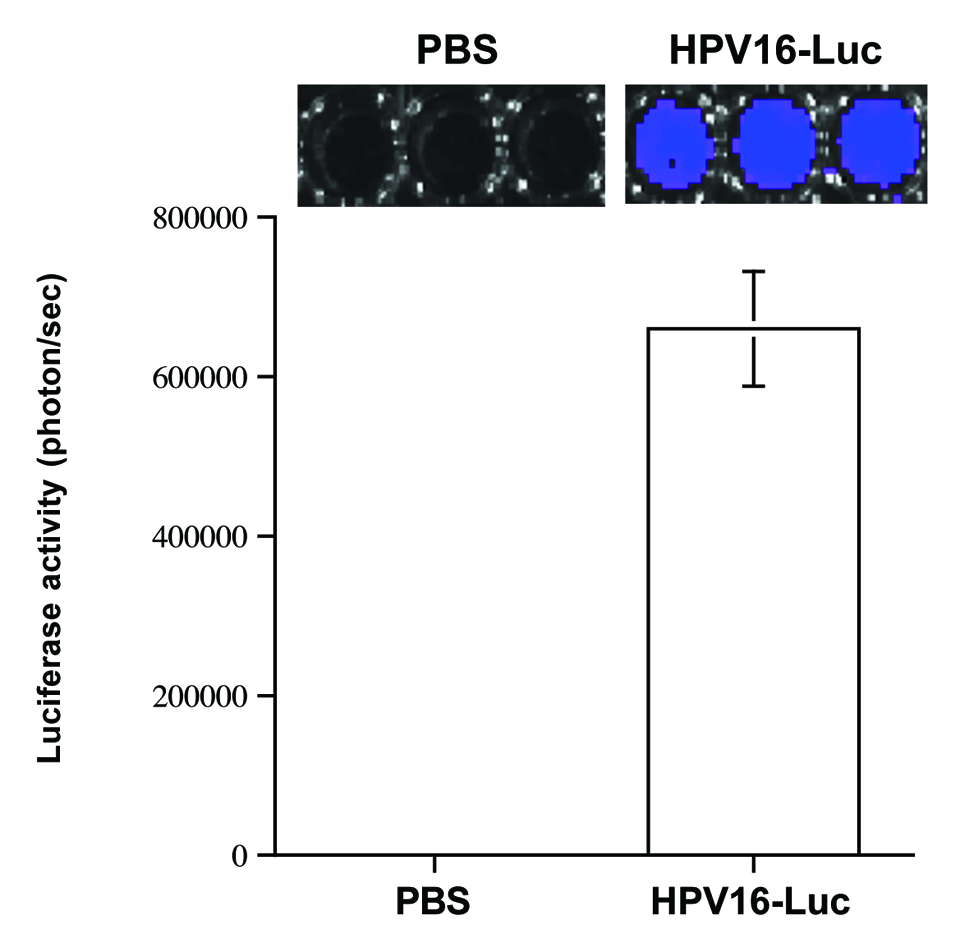

Supplement: Figure S2 — Characterization of the infection of ES2 human ovarian cancer cells by HPV-16 pseudovirions in vitro . ES2 human ovarian tumor cells were seeded into 96-well plates at a density of 5,000 cells/well the night before infection. The seeded ES2 cells were then infected with HPV-16/Luc psV (1 µg L1 protein/ml) for 72 hours and luciferase expression was examined by IVIS 200 system (Xenogen Corp., Alameda, CA, USA). Luminescence images of ES2 ovarian cancer cell line (top). Bar graph depicting the total photon counts for each well (bottom). Note: HPV-16 pseudovirions can efficiently infect ES2 human ovarian cancer in vitro. Data shown are representative of 2 experiments performed. (TIF) [file pone.0040983.s002.tif]

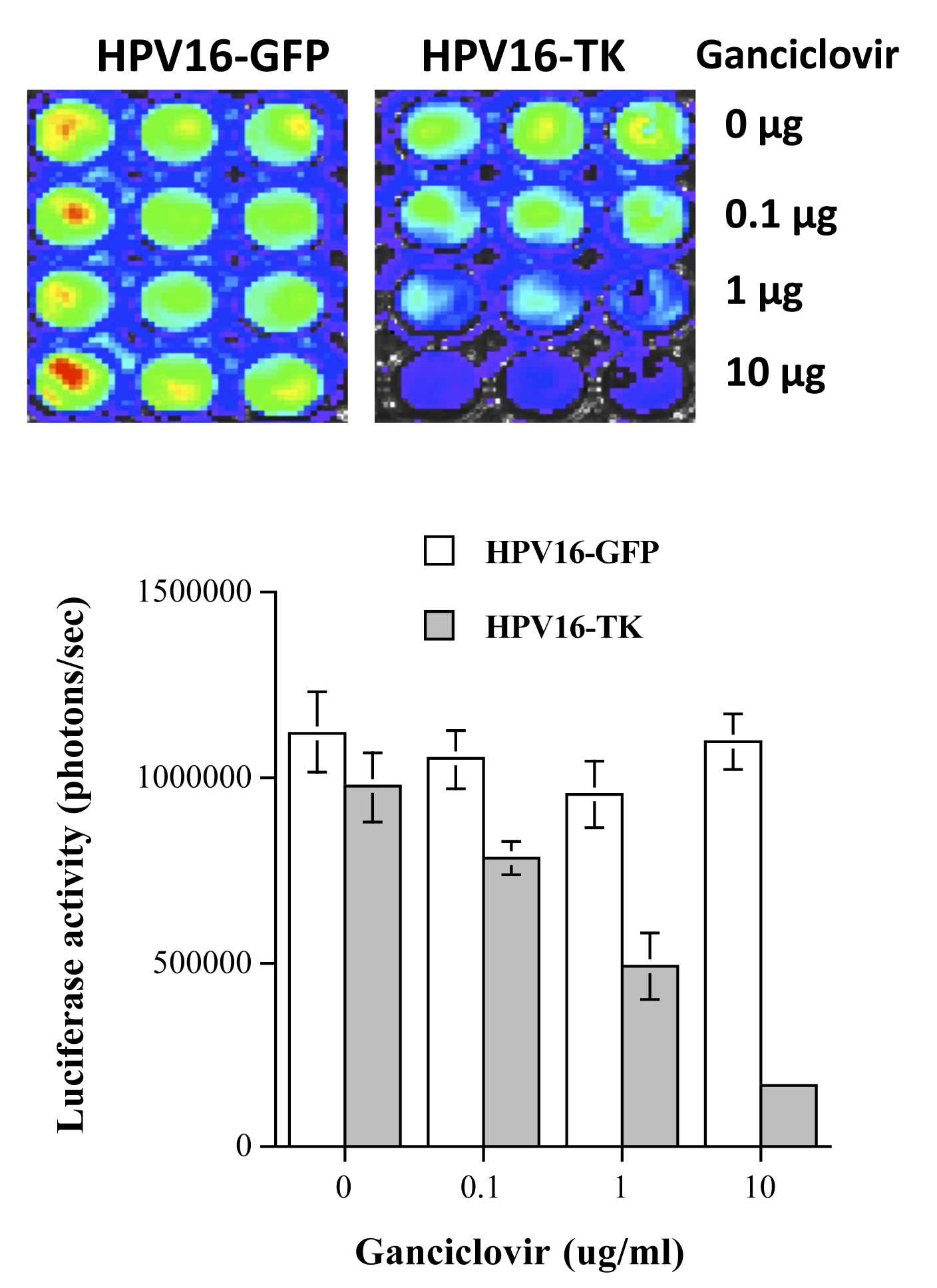

Supplement: Figure S3 — In vitro cytotoxicity mediated by HPV16-tk pseudovirions and ganciclovir. ES2-Luc cells were infected HPV16-GFP psV or HPV16/HSV-tk psV at a concentration of 1 µg L1 protein/ml for 48 hours. The infected cells were seeded in 96 well plates and then treated with 0 µg/ml, 0.1 µg/ml, 1 µg/ml, or 10 µg/ml of Ganciclovir for 72 hours. Luciferase expression was examined by the IVIS 200 system (Xenogen Corp., Alameda, CA, USA). Data shown are representative of 2 experiments performed. (TIF) [file pone.0040983.s003.tif]
